# Supplementary material for: The interactome of intact mitochondria by cross-linking mass spectrometry provides evidence for coexisting respiratory supercomplexes
Source: Mol Cell Proteomics. 2017 Dec 8;17(2):216–32. doi: 10.1074/mcp.RA117.000470 (PMC5795388; doi:10.1074/mcp.RA117.000470)
Supplement: Supplemental Data [file supp_RA117.000470_133922_0_supp_23978_fzffwf.zip › spectra_annotation/mito_DR_spectra_annotation/28-1-1-1-6-1.pdf]

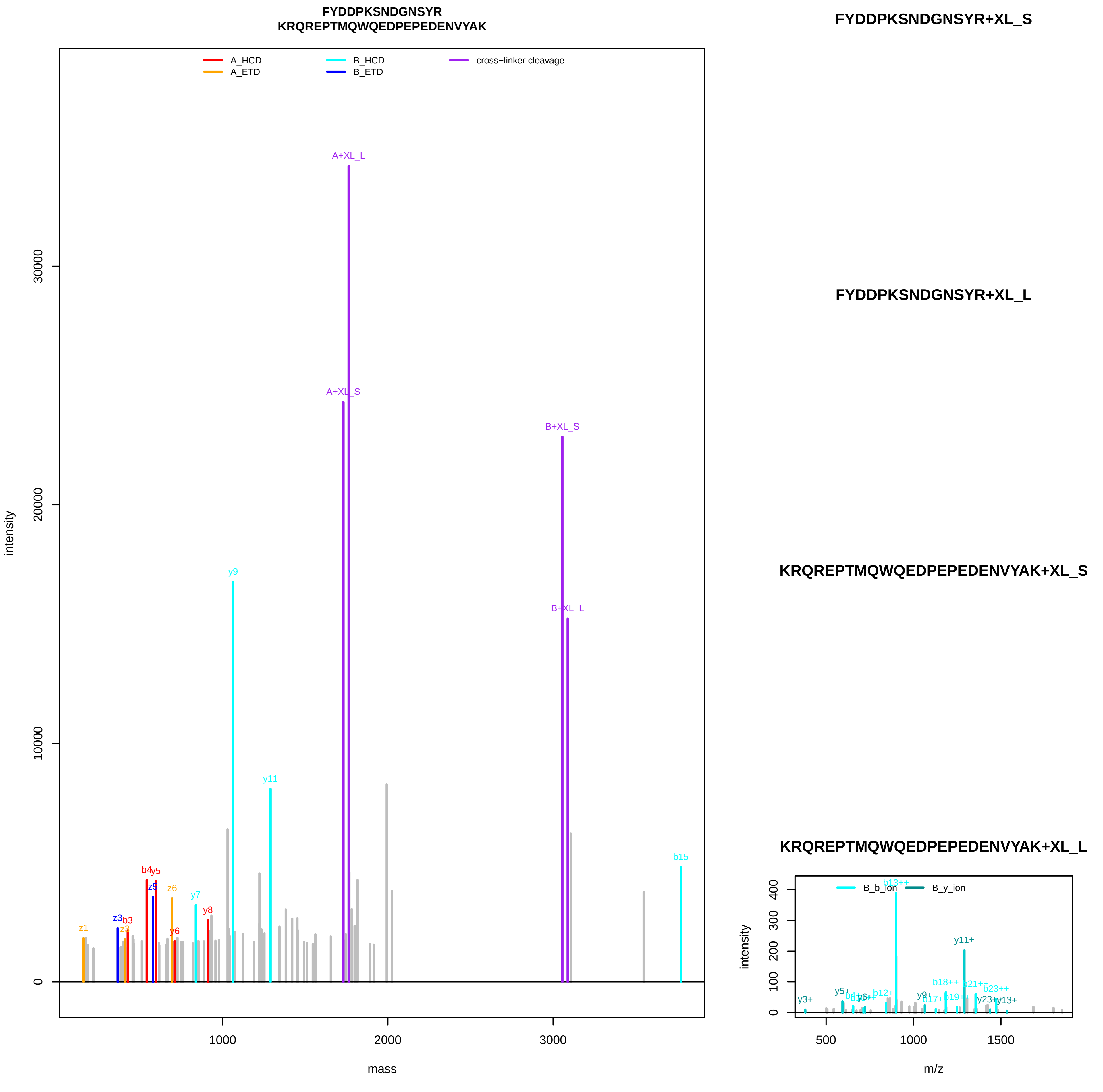
FYDDPKSNDGNSYR
KRQREPTMQWQEDPEPEDENVYAK
A_HCD
A_ETD
B_HCD
B_ETD
cross−linker cleavage
A+XL_L
A+XL_S
B+XL_S
B+XL_L
y9
y11
b15
b4
y5
z5
z6
y7
y8
z3
b3
z2
z1
y6
intensity
mass
0
10000
20000
30000
1000
2000
3000
FYDDPKSNDGNSYR+XL_S
FYDDPKSNDGNSYR+XL_L
KRQREPTMQWQEDPEPEDENVYAK+XL_S
KRQREPTMQWQEDPEPEDENVYAK+XL_L
B_b_ion
B_y_ion
b13++
y11+
y5+
y6+
b12++
y9+
b18++
b21++
b23++
y23+
y13+
y3+
m/z
500
1000
1500
0
100
200
300
400
